# Enhanced extraction of skin interstitial fluid using a 3D printed device enabling tilted microneedle penetration

**DOI:** 10.1038/s41598-021-93235-3

**Published:** 2021-07-07

**Authors:** Sanha Kim, Min Suk Lee, Hee Seok Yang, Jae Hwan Jung

**Affiliations:** 1 Department of Pharmaceutical Engineering, Dankook University, 119 Dandae-ro, Dongnam-gu, Cheonan, 31116 Republic of Korea; 2 Department of Nanobiomedical Science & BK21 FOUR NBM Global Research Center for Regenerative Medicine, Dankook University, Cheonan, 31116 Republic of Korea; 3 Center for Bio-Medical Engineering Core-Facility, Dankook University, Cheonan, 31116 Republic of Korea

**Keywords:** Diagnosis, Laboratory techniques and procedures

## Abstract

Interstitial fluid (ISF) is a body fluid that fills, surrounds cells and contains various biomarkers, but it has been challenging to extract ISF in a reliable and sufficient amount with high speed. To address the issues, we developed the tilted microneedle ISF collecting system (TMICS) fabricated by 3D printing. In this system, the microneedle (MN) was inserted at 66° to the skin by TMICS so that the MN length could be extended within a safe range of skin penetration. Moreover, TMICS incorporating three MN patches created reliable ISF collecting conditions by penetrating the skin at consistent angle and force, 4.9 N. Due to the MN length increase and the patch number expansion, the surface area of the penetrated tissue was increased, thereby confirming that ISF extraction efficiency was improved. Skin ISF was collected into the paper reservoir on the patch, and the absorbed area was converted into a volume. ISF extraction from the rat skin in vivo by TMICS was well tolerated, and the 2.9 μL of ISF was obtained within 30 s. Therefore, TMICS is promising to apply in the diagnosis of multiple biomarkers in ISF with high speed and stability.

## Introduction

Interstitial fluid (ISF), consisting of electrolytes, hormones, and nutrients, is a body fluid that fills and surrounds the cells, providing nutrients to the cells and accepting secretions from the cells^1,2^. Since ISF includes physiological information of the body, it can be utilized as a biomarker source to diagnose various diseases such as diabetes and hyperlipidemia^3–5^. ISF components are similar to plasma and serum and can be directly used for biomarker analysis without pretreatment as an alternative to other body fluids such as blood, urine, and saliva^6^. Moreover, ISF contains various biomarkers that are not present in the blood^7,8^. For instance, exosomes are present in ISF 12–13 times more than blood, so they can be used to diagnose diseases, including cancer^9^. However, conventional methods for ISF extraction, such as a suction blister, depth-limited hypodermic cannula, and open-flow microperfusion, are time-consuming, risky to patients, demanding medical expertise and specialized instrument^10–12^.

To address the issues, microneedles (MNs) have been proposed to extract skin ISF with a painless and minimally invasive strategy. MNs are fabricated from metal or polymer with hundreds of microns in length and penetrate the main barrier of the skin, stratum corneum, thereby creating a pathway accessible to skin ISF^2,13,14^. The style of MN can be designed depending on the applications. Since the hydrogel MNs can release the skin ISF out again, it is advantageous to use when an additional reaction with other reagents or analysis by external instruments is needed^13–16^. On the other hand, when a rapid on-site diagnosis is needed, ISF extraction using metal MNs is advantageous. Typically, the metal MNs are incorporated with a piece of paper where biomarkers can be detected^3,7,17–19^. As the surface of metals such as steel and stainless steel is hydrophilic, once it penetrates the stratum corneum, it attracts skin ISF and allows it to wet across the surface of the metal MNs, transferring ISF to the papers. The biomarker from ISF delivered to the paper is immediately detected, reducing the possibility of damage and sample contamination and saving detection time^20^. Moreover, most of the extracted ISF can be used for diagnosis, thereby improving detection sensitivity. Paper-based diagnostic platforms are simple, inexpensive, and can check most detection signals at a glance. For example, a lateral flow strip can detect a target such as food poisoning bacteria with the naked eye by coloring reaction without expensive detection equipment^19,21,22^. Most of all, metal MNs including the paper reservoir, were reported to extract ISF at a rate of 2–4 μL per minute under in vivo conditions, which is faster than that of hydrogel MN^3,13–16^.

It is essential to improve the diagnosis sensitivity by securing the stability of biomarkers and a sufficient quantity of samples to minimize diagnostic errors such as false positives and false negatives^23,24^. Therefore, MN devices for biomarker diagnosis should have the ability to obtain enough amount of ISF within a safe range with rapid extraction speed. Rapid ISF sampling increases sample integrity and patient compliance, and collecting a sufficient sample expands the range of applicable biomarkers. A simple way to improve ISF extraction capacity from the metal MN is to expand the area of the MNs contacting the tissue. However, when the width of MN increases, the patient's pain increases, and the skin permeability decreases, while increasing the length allows the needle to penetrate the capillaries of the upper dermal layer and also increases the patient's pain^25,26^.

To solve these limitations, we developed a Tilted microneedle ISF collecting system (TMICS) that can extract large amounts of ISF at high-speed using 3D printing technology. In this system, the MN length was extended to increase the contact area between the MN and skin tissue, while the MN was inserted at an angle to the skin by TMICS for minimal skin penetration. TMICS was designed using computer-aided design (CAD) software and was manufactured through a 3D printer to realize accurate and reproducible insertion angles. Thus, the metal MNs incorporated in TMICS were inserted by the same force and angle, and three metal MN patches were integrated into a single TMICS to increase ISF collection. To prove that the tilted MN insertion is efficient for ISF extraction, a straight MN ISF collecting system (SMICS), which has the same skin penetration depth as TMICS, was fabricated and tested. Furthermore, the efficiency of TMICS were analyzed in animal models.

## Results

### Fabrication of the MN devices using 3D printing

**Figure 1:**
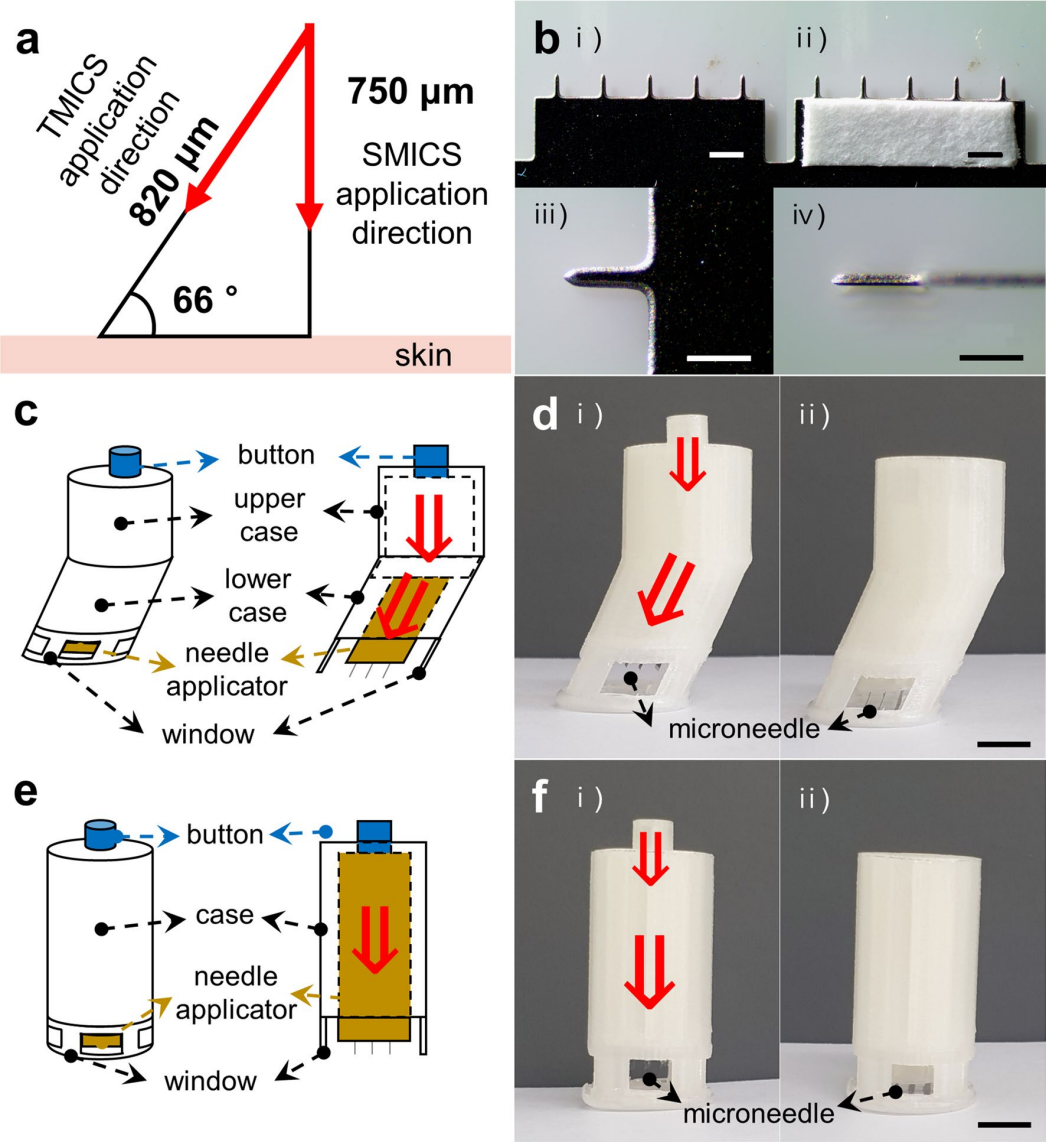
Information about TMICS, SMICS, and MN. (**a**) A schematic diagram showing information of TMICS: The insertion angle is 66° and the penetration depth is 820 μm. (**b**) Optical images of a stainless microneedle array (i) and the MN array attached a paper reservoir (ii). The scale bars in (i) and (ii) are 1 mm. The magnified images of a microneedle top view (iii) and side view (iv). The scale bars in (iii) and (iv) are 500 μm. (**c**) Schematic illustration of TMICS. (**d**) Digital images of TMICS before MN application (i) and after MN application (ii). The scale bar in (**d**) is 1 cm. (**e**) Schematic illustration of SMICS. (**f**) Digital images of SMICS before MN application (i) and after MN application (ii). The scale bar in (**f**) is 1 cm.

Stainless MN patch was designed by 3D max and customized from SKB Tech (Ulsan, Korea). The MN length combined in TMICS was calculated to 820 μm considering the previous MN length of 750 μm used in the previous studies^1,18^ and the MN insertion angle (66°) so that to obtain the same skin penetration depth between the 820 and 750 μm MNs (Fig. 1a). The customized MN patch (SKB Tech, Ulsan, Korea) consists of five MNs with 820 μm length, which is measured 50 × 200 μm in cross-section and tapered to a tip with less than 1 μm radius (Fig. 1b,i–iii). A paper reservoir (Whatman grade 1, Sigma) with 2 × 7 mm was attached on both sides of the MN to store extracted ISF (Fig. 1b,iv). The MN devices, TMICS and SMICS, were designed by 3D max (Fig. 1c,e, Supplementary Figs. S1, and S2) and fabricated using PLA filaments by a Fused deposition modeling (FDM) 3D printer (M160, Moment, Seoul, Korea) (Fig. 1d,f). To enhance the resolution of the 3D printer, we changed the temperature range of the heating bed 60, 70, 80 °C with maintaining the heating bed at 230 °C (n = 3). TMICS and SMICS were fabricated at high resolution without defects or cracks in all the given temperature conditions (Supplementary Fig. S3).

### Ex vivo skin penetration test using the MN devices

To measure the minimum force that TMICS and SMICS need to insert the MNs into the skin, we placed a weight of 200 g, 500 g, or 1 kg on the button of the devices. In TMICS, when a 200 g weight was used, the button stayed the place (Supplementary Fig. S4a,i). While when a 500 g weight was placed on the button, it pushed the button downward (Supplementary Fig. S4a,ii). In SMICS, when a 500 g weight was used, the button was not pressed, but it moved the button when a 1 kg weight was used (Supplementary Fig. S4b,i,ii). As a result, it was found that the forces of 4.9 N and 9.8 N were required to operate TMICS and SMICS, respectively, and that TMICS used less power to insert microneedles (n = 3).

**Figure 2:**
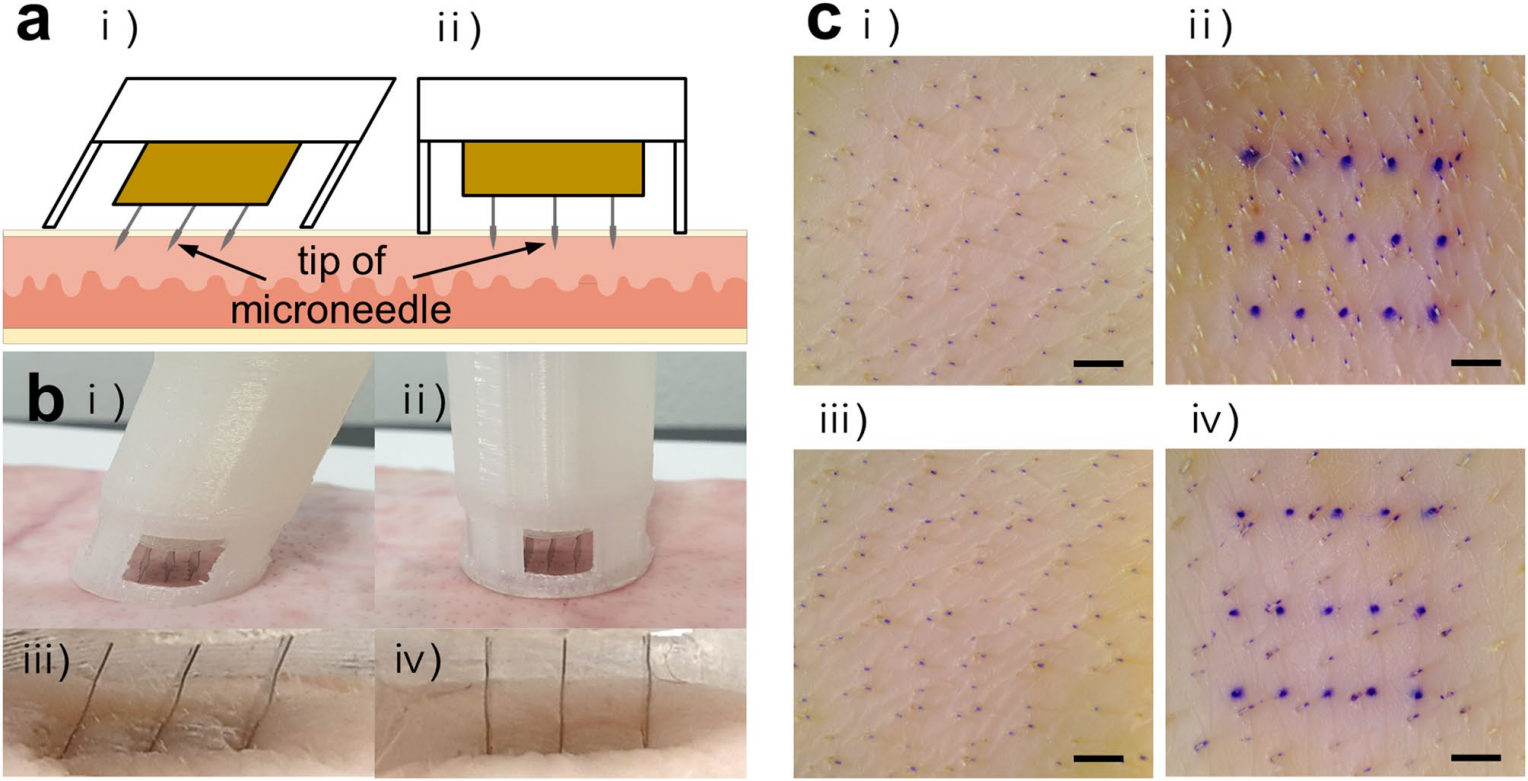
Ex vivo skin penetration test using the MN devices. (**a**) Schematic illustration of the MN insertion into a porcine skin ex vivo by TMICS (i) and SMICS (ii). (**b**) Digital images of the MN penetration by TMICS (i) and SMICS (ii). TMICS MNs penetrated the skin with a tilted state (iii), and SMICS MNs penetrated vertically (iv). (**c**) Optical images of the skin before (i and iii), and after skin penetration by TMICS (ii) and SMICS (iv). The scale bars are 1 mm (n = 3).

A skin penetration test was performed using the MN devices on the porcine skin ex vivo (Fig. 2a). When we applied TMICS, the MNs have successfully penetrated the skin in a tilted state, and it was confirmed via the window (Fig. 2b,i,ii). The MNs of SMICS penetrated the skin vertically and were shown through the window (Fig. 2b,iii,iv). To prove that the MNs penetrate at a designated angle, the angle of the MN inserted into the PDMS artificial skin was measured. The insertion angle of TMICS and SMICS were measured at 66.3° ± 0.8° and 90.2° ± 0.9°, respectively (Supplementary Fig. S5). After application of both TMICS and SMICS, the treated skins were stained with a gentian violet solution (n = 3) (Fig. 2c). As the results, it was confirmed that both MN devices could stably allow the MNs to penetrate the skin and be used as devices for ISF extraction (Fig. 2c,ii,iv).

### Measurement of ISF volume using a filter paper

**Figure 3:**
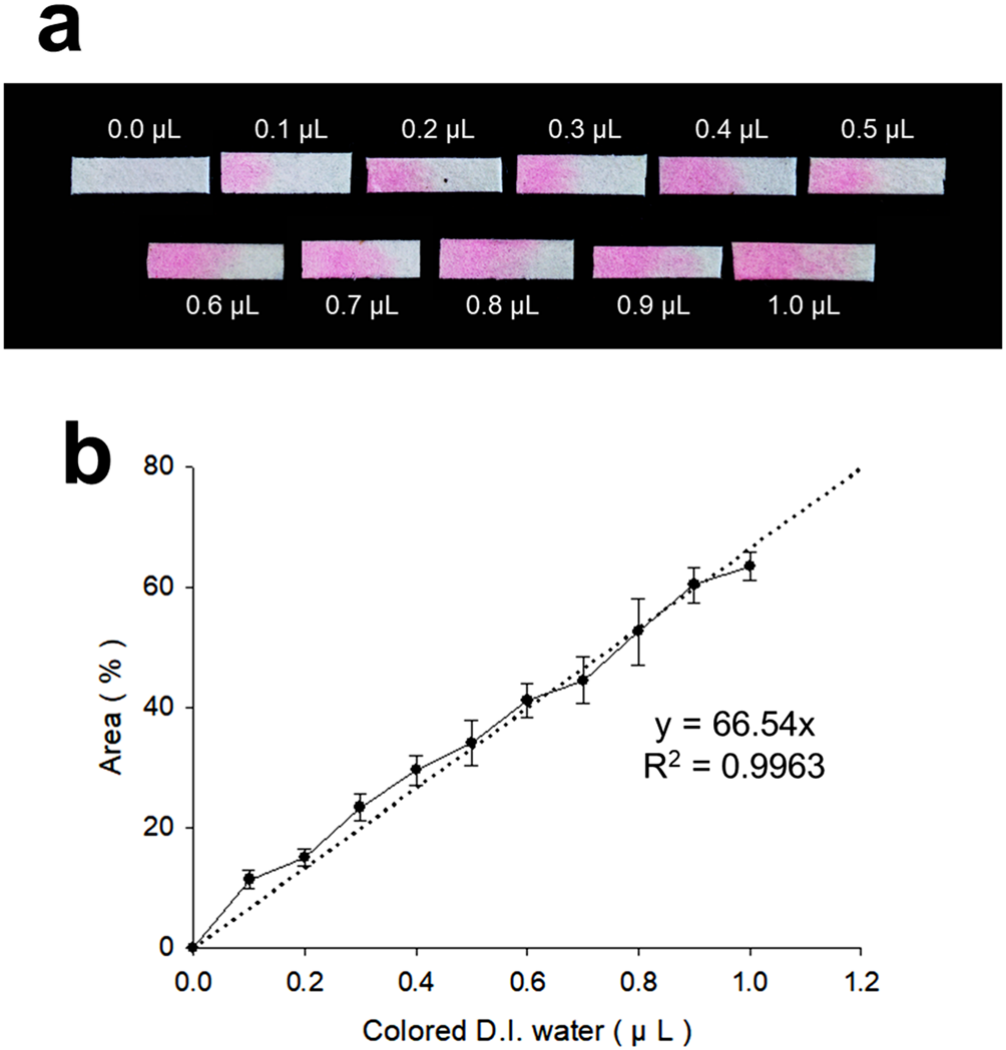
Quantification of a pink dye solution using the paper reservoir. (**a**) Digital image showing that as the solution volume increases to 0–1 μL at 0.1 μL intervals, the area of the solution in the paper storage is increased. (**b**) A graph showing the linearity of the solution volume and the ratio of the area occupied by the solution. Graph b presents average ± standard deviation based on 5 replicate samples.

The extracted ISF was quantified by measuring the paper reservoir area occupied by the pink dye solution (Fig. 3a). As the volume of a pink solution increased, the area of the paper reservoir also increased. The solution volume was linearly correlated to the area occupied by the solution to the area of the entire paper reservoir (Fig. 3b). The relation between the solution and the area ratio was obtained to y = 66.54x, and the R^2^ value was 0.9963, proving the perfect linearity. Therefore, we have demonstrated that ISF extracted from skin can be quantified as the paper reservoir based on these results.

### ISF collection from a porcine skin ex vivo

**Figure 4:**
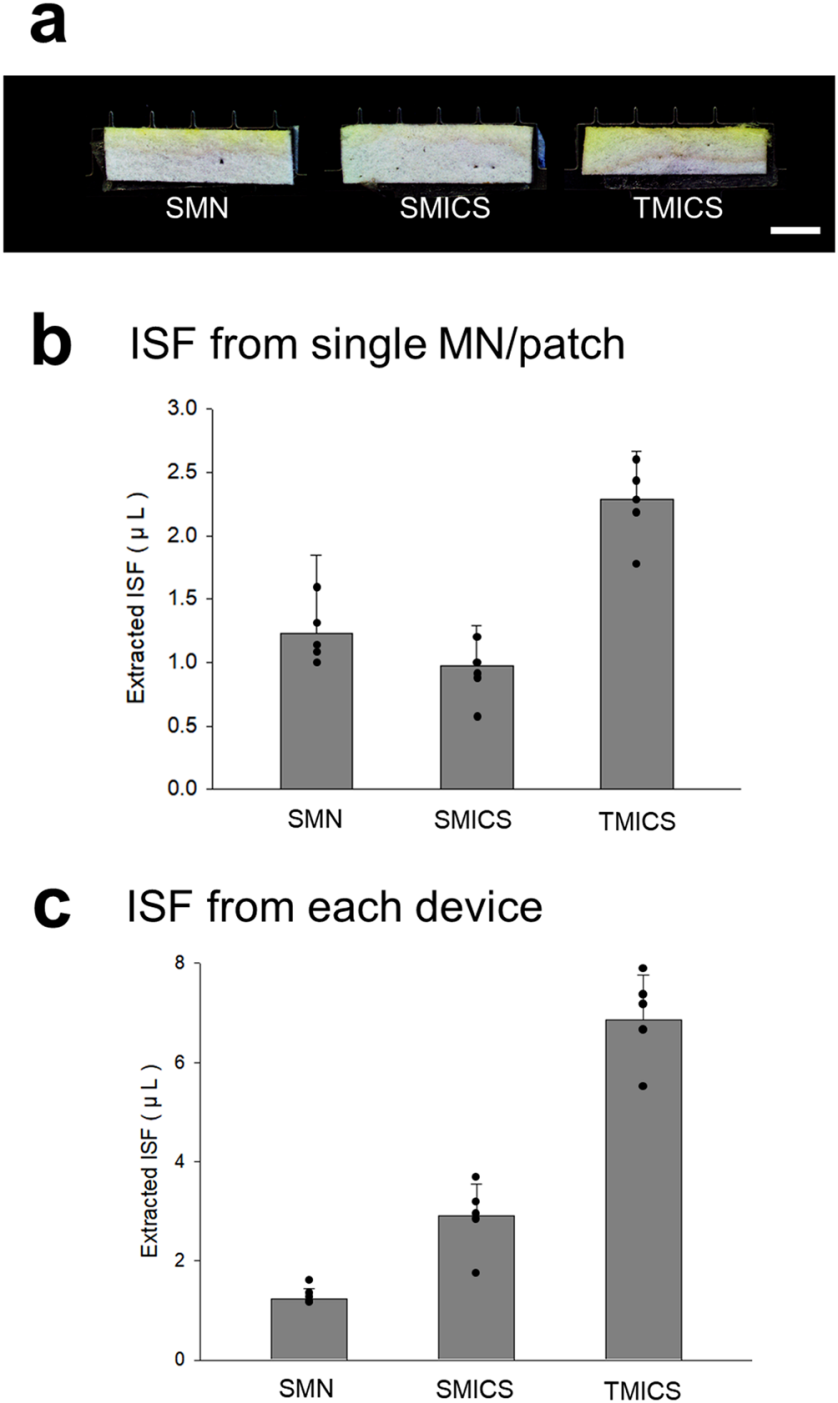
ISF collecting using the MN devices from a porcine skin ex vivo. (**a**) Digital image of the MN devices, SMN, SMICS, and TMICS, after collecting skin ISF. (**b**) Extracted skin ISF from a single MN patch (both side) of the MN devices. (**c**) Total extracted ISF from the MN devices. Graphs present average ± standard deviation based on 5 replicate samples (one-way ANOVA, p < 1.7 × 10^–5^).

ISF extraction was performed to the porcine skin ex vivo using TMICS and SMICS. To determine the wet area of the paper reservoir by ISF extraction, the porcine skin was incubated in the fluorescein solution overnight before the extraction. ISF extraction was performed using a Single MN (SMN) patch, SMICS, and TMICS, the extraction efficiency of those devices was analyzed and compared. ISF, including fluorescein, was extracted to the paper reservoir with yellow-green color from the porcine skin ex vivo (Fig. 4a). ISF was wet evenly from the MN side, and the boundary of the wicking area on the paper was shown. When comparing the wicking area of ISF extracted from a single MN of each MN device, we could confirm that TMICS collected more ISF than an SMN and SMICS as expected. The portion (%) of the wet area occupied by ISF to the entire paper reservoir was converted to the volume based on the obtained relation (Fig. 3b). Then, the extracted ISF volume per MN patch was plotted depending on the MN devices (Fig. 4b). SMN patch (without the device) could collect 1.22 ± 0.66 μL of ISF, and SMICS and TMICS extracted 0.97 ± 0.32 and 2.28 ± 0.39 μL of ISF, respectively (one-way ANOVA, p < 1.7 × 10^–5^).

When ISF was extracted with TMICS, the extracted amount was increased by 2.4 times that of SMICS. Comparing the skin contact area of the MNs used to TMICS and SMICS, the contact area increased by 9.5% when TMICS was used than SMICS. It means that the contact area of 0.0185 mm^2^ per MN, and 0.0925 mm^2^ per patch (five MNs) increased. As a result, compared to the increase of the penetration surface area, the amount of ISF extraction increased dramatically, and it can be seen that widening the surface area is critical for increasing the amount of ISF extraction.

SMN (without the device) extracted more ISF than a single MN patch of SMICS (Fig. 4b). The reason is that since SMICS has a constant insertion angle of 90° controlled by the 3D printed device, the contact area of the MNs to the skin is minimized. On the other hand, the SMN performed by hands has an inconsistent insertion angle, increasing the MN contact area and ISF extraction. When analyzing the extraction amount of each MN device, the error range of SMN (1.22 ± 0.66 μL) is larger than those of SMICS and TMICS (0.97 ± 0.32 and 2.28 ± 0.39 μL). Therefore, the results prove that ISF extraction by the 3D printed device creates a uniform ISF sampling environment by inserting the MNs with the same penetration angle and force.

SMICS and TMICS, both containing three MN patches, could collect ISF with 2.91 ± 0.48 and 6.86 ± 0.42 μL, which are 2.4-fold and 5.6-fold bigger than the SMN patch (1.22 ± 0.66 μL) (one-way ANOVA, p < 1.2 × 10^–7^) (Fig. 4c). The more MN patches, the more ISF extraction amount increased in proportion to that, and the 3D printed device contributed to the expansion of the MN patches. Since the total application time of the device was 30 s, considering the interval between MN applications, it was confirmed that the maximum ISF of 13.7 μL/min could be extracted through ex vivo ISF collecting. Moreover, when TMICS was used, the extraction amount increased by threefold than SMICS. Consequently, we demonstrated that an increase in the contact area to the skin, controlled by the MN insertion angle and number of MNs, is critical for ISF extraction.

### ISF collection from a rat skin in vivo

**Figure 5:**
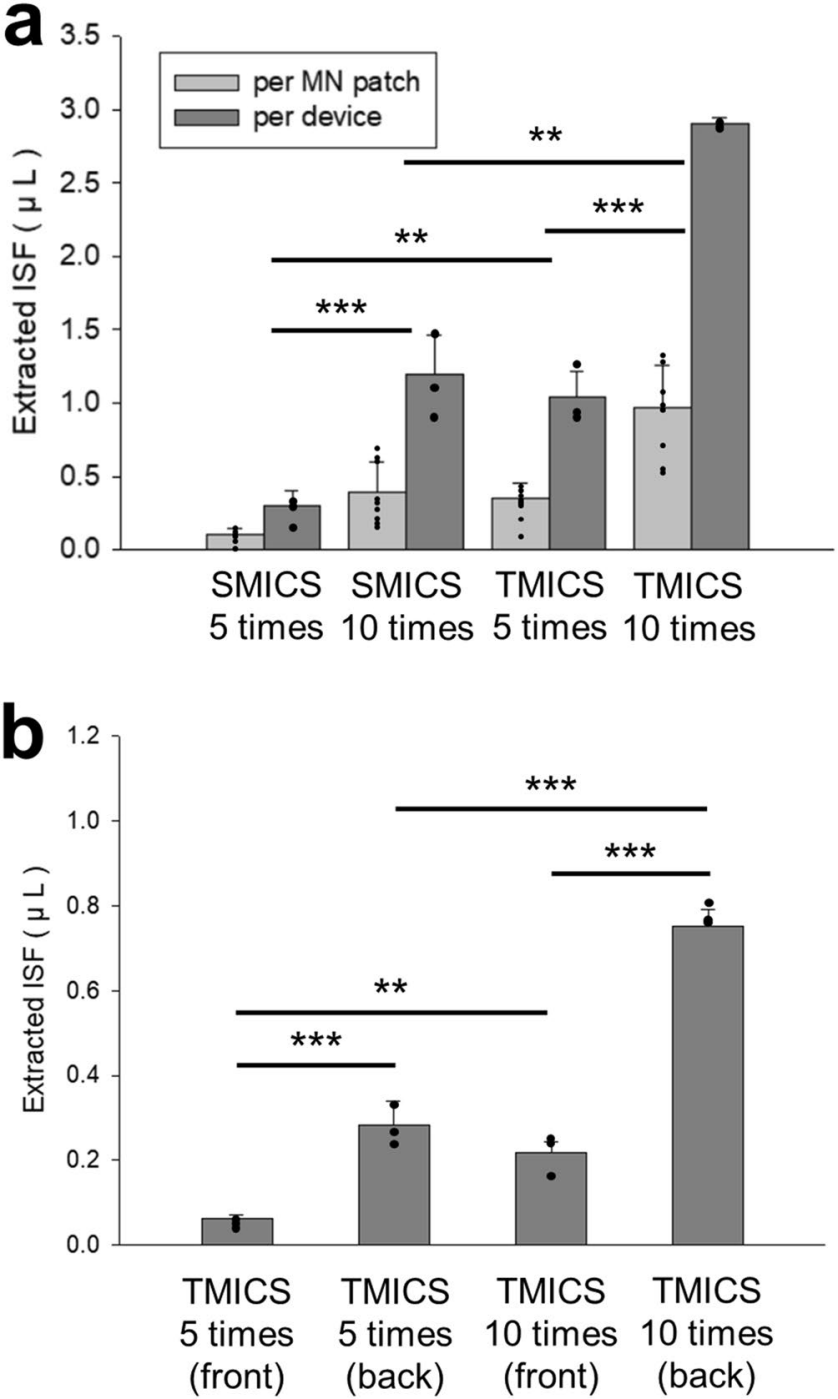
ISF collecting using the MN devices from the skin of Wistar rat in vivo. Skin ISF was extracted from SMICS or TMICS with five- or ten-times application. (**a**) Total extracted ISF from the front and backside of the MN patch (per each MN patch and also per each device). (**b**) Extracted ISF from the front and backside of the MN patch in TMICS with five- or ten-times application. Graphs present average ± standard deviation based on 3 replicate samples. **,***Indicate significant difference (An unpaired t-test, p < 0.005, 0.001, respectively).

Based on the ex vivo results, we studied ISF extraction efficiency using TMICS on the rat skin in vivo. To optimize the collecting condition, we controlled the MN insertion number to five- or ten-time (Fig. 5). SMICS collected 0.3 μL (0.10 ± 0.05 μL/patch) at a five-time insertion and 1.2 μL (0.40 ± 0.20 μL/patch) at a ten-time insertion (unpaired t-test, p < 0.00001) (Fig. 5a). On the other hand, TMICS extracted 1.0 μL (0.35 ± 0.10 μL/patch) at a five-time insertion and 2.9 μL (0.97 ± 0.29 μL/patch) at a ten-time insertion (unpaired t-test, p < 0.0007). The results demonstrated that the ten-time insertion of ISF extraction devices was four-fold more efficient in SMICS and 2.9-fold more efficient in TMICS than the five-time insertion of the devices. Comparing the MN devices, TMICS was 3.3-fold and 2.4-fold more efficient at the five- and ten-time insertion than SMICS, respectively (unpaired t-test, p < 0.0011 and p < 0.0013). These results clearly show that ISF extraction is increased by the pumping effect caused by the punctures generated from the repeated MN insertions and the motions caused by the repeated MN application to the same position^1,18^. Although slight bleeding was observed during ISF extraction, there was no significant error among sample volumes, and ISF was successfully collected in the paper reservoir (Supplementary Fig. S6). As a result, ISF extraction volume increases as the number of MN insertions increases.

Since TMICS is inserted into the skin at an angle of 66°, we expected that there would be a difference in the amount of ISF extracted back and front from the patch (Fig. 5b). When the device was applied five times, the front side of the patch extracted 0.06 ± 0.05 μL of ISF, while the backside collected 0.28 ± 0.11 μL of ISF, 4.67 times more than the front (unpaired t-test, p < 0.00007). In the ten-time applications, the front side extracted 0.22 ± 0.10 μL of ISF, and the backside collected 0.75 ± 0.21 μL of ISF, 3.41 times higher than the front side (unpaired t-test, p < 0.000007). In all tests, the backside of the patch collected more ISF simultaneously, which indicates since the backside of the patch comes into close contact with the skin as the device is inserted at an angle to the skin (n = 3 rats). Therefore, it is essential to place the paper reservoir in direct contact with the skin when inserting the MN, as even small differences in the paper reservoir location will affect ISF collection.

### Histology analysis

**Figure 6:**
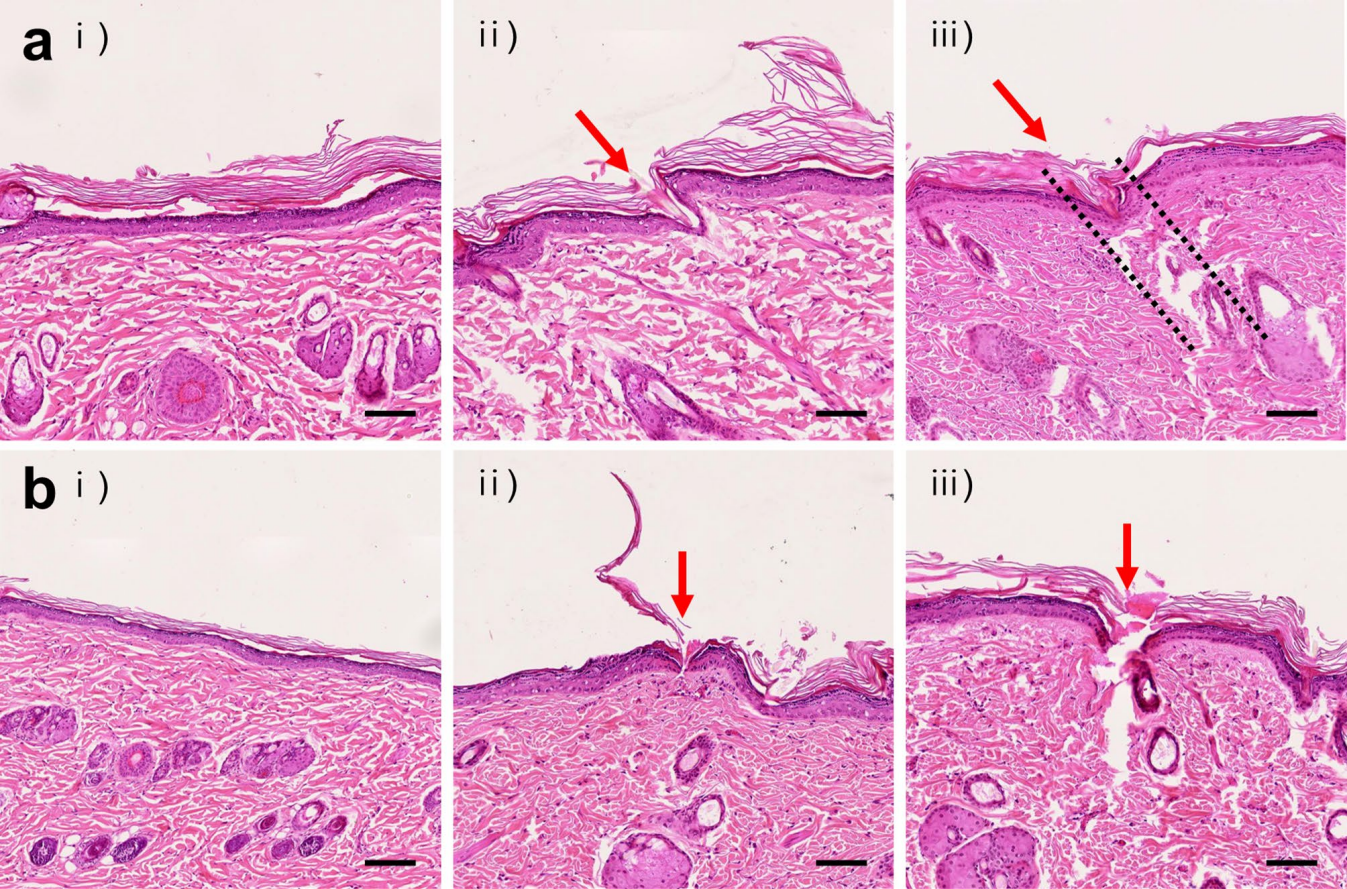
Histological images of rat skin. Representative histological images of rat skin tissues after application of TMICS (**a**) and SMICS (**b**). (i) Untreated skin (Control), (ii) skin poked five times, and (iii) skin poked ten times with the devices. Red arrows [in (ii), (iii)] and black dashed lines [in **a**(iii)] indicate the trace of MN penetration. All scale bars are 100 μm. (n = 3 rats).

A histology analysis was performed to verify the difference in MN insertion according to the device and to confirm the damage to the skin tissue due to the repeated device application (Fig. 6). In histology samples, the differences between the control and the MN-applied tissues were confirmed. However, no skin tissue damage was found due to MN penetration. The trace of MN penetration was determined by confirming the damage of the stratum corneum and epidermal layer. In the tissue applied five-time, the MN trace was found less than that applied with the ten-time (Fig. 6a,ii,iii,b,ii,iii). When the tissue applied ten-time was observed, the tissues treated by TMICS were penetrated by the MN in a tilted shape, and SMICS was vertically poked (Fig. 6a,iii,b,iii). Therefore, the histology analysis showed that the repeated MN insertion for ISF collection using TMICS and SMICS was well tolerated without damaging the tissues.

## Discussion

To increase the detection accuracy of biomarkers present in ISF, it is essential to secure reliable and uniform sample quantity. However, since the MN patches reported in the previous studies are applied to the skin by hands directly, the insertion angle and force and the amount of ISF extraction may vary at every application^3,7,17,18^. Therefore, we designed the 3D printed devices, TMICS and SMICS, to secure a stable and uniform amount of ISF and penetrate MNs into the skin at the same force and angle. As a result, it was confirmed that the force of 4.9 N and 9.8 N was required to operate TMICS and SMICS, respectively (Supplementary Fig. S5), and histology analysis confirmed that TMICS makes the MNs insert at an angle and SMICS vertically into the skin (Fig. 6). Therefore, we have demonstrated that the insertion angle and force of the MNs for ISF extraction were adjusted consistently using the 3D printed devices.

This study was conducted to secure a sufficient volume of ISF by varying the insertion angle of the MNs. The simplest method for collecting as much ISF as possible among tissues is to increase the MN length to widen the contact area between the MN and the skin tissue. However, in that case, the penetration depth of the MN is deepened, thereby causing pains. To overcome the issues, we inserted the MNs with a tilt at 66° into the skin to increase the contact area between MNs and tissues while minimizing the penetration depth. By fabricating TMICS by 3D printing, MNs were penetrated at a constant angle of 66°. SMICS was fabricated to insert MNs vertically with the same penetration depth as TMICS, thereby confirming how the insertion angle affects ISF extraction. Consequently, when TMICS was inserted into rat skin in vivo ten times (for 30 s), the extraction efficiency was 2.4 times better than when SMICS was applied under the same conditions (Fig. 5). Therefore, we confirmed that increasing the contact area by inserting the MN at an angle facilitates ISF extraction, and the 3D printed device consistently adjusted the MN insertion angle.

Expansion of the MN patches is a simple way to increase the amount of ISF extraction. This study incorporated three MN patches on the 3D printed device to enhance ISF extraction (Figs. 2 and 5a). As the patch is expanded like TMICS and SMICS, we can secure diagnostic reproducibility by detecting the same target multiple times in a single device and diagnostic diversity by simultaneously detecting numerous biomarkers in a single device. According to the in vivo study results, since 1.0 μL/min of ISF is extracted per patch, a simultaneous detection system for targets such as glucose, cholesterol, or vancomycin can be implemented^27–29^. Since this study focused on finding the optimal ISF extraction conditions and securing a sufficient ISF, the detection of biomarkers in ISF was not conducted. However, based on this study, we plan to perform a target diagnosis study using TMICS or an improved device.

To increase ISF extraction and obtain a consistent volume of ISF, we fabricated the 3D printed devices (i.e., TMICS and SMICS), which are combined three MN patches, and conducted a study that penetrated the skin with accurate force and angle. When the MN was inserted at an angle of 66° using TMICS, a high ISF extraction rate of 5.8 μL/min could be obtained from rat skin in vivo (Fig. 5b). Compared with the extraction rate < 4 μL/min of previously published studies^3,17,18,20^, it was confirmed that ISF extraction of TMICS was significantly improved. Although the previous studies obtained the extraction rate from a single MN patch consisting of nine MNs, considering that three MN patches consisting of five MNs are combined in TMICS, it can be seen that the extraction rate of TMICS does not improve significantly^3,17,18^. To increase the efficiency of TMICS, further research is required through modification and supplementation of the device. First, increasing the number of MNs or combining more patches to TMICS can increase the pathway required for ISF extraction. Moreover, by lowering the MN insertion angle, the depth of invasion is reduced, and the MN length can be extended to widen the contact area between the needles with the tissue, thereby improving ISF extraction. Applying heat within a safe range around the skin to which the patch is applied or physical stimulation such as massage to promote ISF circulation can also increase the extraction efficiency^1^.

## Conclusion

In this study, we studied a method for uniformly extracting large amounts of ISF using a MN patch. Using 3D printing technology, TMICS that can penetrate three MN patches simultaneously through the skin with the same force was fabricated. Furthermore, TMICS was designed so that the MN can be inserted at an angle of 66° to minimize skin penetration and pain and to widen the contact area between the tissue and MNs. It was confirmed that TMICS penetrated the skin with a force of 4.9 N and a specific angle through the ex vivo skin penetration test, and 2.9 μL of ISF was extracted within 30 s through in vivo study and histology analysis. Moreover, the reliable condition for ISF extraction was created using a 3D printed device, thereby extracting uniform ISF. Since TMICS consists of three MN patches, we also showed the possibility of simultaneously detecting multiple biomarkers with a single ISF extraction in future research. Therefore, TMICS is promising to construct a patient-friendly disease diagnosis and monitoring system by applying biomarkers in various ISFs.

## Experimental

### Materials and methods

Filter paper (Whatman grade 1), gentian violet solution (2%), phosphate-buffered saline, fluorescein, paraformaldehyde (PFA, 4%), hematoxylin–eosin, and isoflurane were purchased from Sigma-Aldrich Korea (Seoul, Korea). Porcine skin ex vivo (3 × 3 cm) was purchased from Cronex (Seoul, Korea). Xylene was obtained from Daejung (Gyeonggi-do, Korea). All methods were carried out in accordance with relevant guidelines and regulations.

### Fabrication of the tilted MN ISF collecting system

TMICS is a device for efficiently extracting skin ISF by injecting MNs with the same force as the exact slope. It consists of a button, upper and lower case, MN holding part, stainless MNs, and windows (Fig. 1c). We designed the lower case to have a slope of 66° so that the force applied vertically to the button is transmitted to the skin by diagonal as like the direction of the red arrow (Fig. 1c,d). The angle of 66° is designed to safely facilitate holding and applying the device with one hand on the skin. When the button is pressed with 4.9 N, the power is transferred to the MN holding part through cylinders located in the upper and lower case. Then, the MN patches combined under the holding part move to penetrate the skin. The exact penetration of the MNs can be confirmed through the windows of the apparatus most bottom (Fig. 1d). The total length of the assembled device is 52 mm, and the MN holding part is combined with three MN patches, which contains a total of 15 needles (5 MNs × 3 patches) per TMICS.

To compare ISF extraction of TMICS, we fabricated a straight-type MN ISF collecting system (SMICS), which was designed to have the same height and similar configuration as TMICS (Fig. 1e). The device is designed to allow the MNs to penetrate the skin vertically by pressing the button with 9.8 N (red arrow in Fig. 1e,f), and the height of the MN holding part is adjusted to make the same penetration depth (i.e., 750 μm) as when the MN of TMICS is inserted into the skin at 820 μm and 66° (Fig. 1f).

The components of TMICS and SMICS were designed by CAD program (Supplementary Figs. S3 and S4), and all parts except the stainless MNs were output by an FDM 3D printer which is inexpensive and has an appropriate resolution for manufacturing TMICS and SMICS. Poly-lactic acid with hard properties was used as a 3D printing material, and each component was assembled using an acrylic adhesive after output (Fig. 1d,f).

### Measurement of ISF volume using a filter paper

Typically, the extracted ISF is a small amount of 2–4 μL per minute or less and can quickly evaporate and lose under ambient conditions, so it is not easy to accurately measure the extracted ISF. To facilitate the measurement of ISF extraction, the relationship between the wet area of the paper reservoir and the amount of ISF extracted was derived. A pink dye solution in PBS buffer was dropped by a micropipette to the paper reservoir, which is the same material and size (2 × 7 mm size) used for the stainless MN patch, and we analyzed the wet area of the paper reservoir via ImageJ. However, to accurately measure the extracted ISF, quantification must be possible in hundreds of nanoliters, which exceeds the measurement limit of micropipettes. Therefore, we expanded the paper reservoir area four times (4 × 14 mm), experimented with it, and then converted it back to the original size (2 × 7 mm). Consequently, the extracted ISF volume was calculated by measuring the area of the dye solution wet on the paper reservoir at 0.1 μL intervals in the range of 0 ~ 1 μL (n = 5).

### Skin penetration test using TMICS

The skin penetration by TMICS was performed using a porcine skin ex vivo. The skin was prepared by removing furs and cleaning with an alcohol swab and was firmly fixed on the board by pins. Then, we poked the skin using TMICS and put 2% of the gentian violet solution on the treated skin for 10 min. After removing the gentian violet, we took the skin image using a microscope (Olympus, SZ61 TR, Tokyo, Japan) to check the skin penetration by TMICS.

### ISF collection from a porcine skin ex vivo

The porcine skin ex vivo (3 × 3 cm) was cleaned with an alcohol swab and then incubated in the 0.1 mg/mL of fluorescein solution in PBS overnight. Before applying TMICS, the fur of the skin was removed by a razor and cleaned using an alcohol swab. After drying the skin surface, we applied TMICS ten times with 3-s intervals (for 30 s) to collect ISF. The wet area of the paper reservoir by fluorescein was taken using a microscope (Olympus, SZ61 TR, Tokyo, Japan), and the extract ISF volume from the wet area was obtained by analysis of ImageJ. All the experiments were performed at least five times.

### ISF collection from rat skin in vivo

Wistar rats (8 weeks old, male, 180–200 g weight, RAONBIO, Gyeonggi-do, Korea) were anesthetized by isoflurane (Forane, Chongwae Pharma, Seoul, Korea) inhalation during all procedures. Before one day of the experiment, the back of rat hair was removed using an electric razor, and then a depilatory cream (SEWHAP&C, Chungcheong-do, Korea) was used to remove the rest of the short hair of the skin. After cleaning the skin, TMICS, SMICS, or Single MN patch was applied ten times with a 3-s interval for 30 s to extract skin ISF in vivo (n = 3 rats). The MN devices were consistently applied to the same part of the rats, upper back. To confirm the change of ISF extraction amount according to the application number of the MN devices, TMICS and SMICS were applied 5 and 10 times each. After the device application, the skin tissues, including the panniculus carnosus layer, were collected using a surgical scissors and were inserted between two slide glasses then sealed by parafilm. The untreated control skins were collected from the other side of the treated rats. Then, the skin was fixed with 4% paraformaldehyde (PFA, Sigma-Aldrich). All procedures related to animal experiments were carried out under a protocol approved by the Institutional Animal Care and Use Committee of Dankook University (Approval No. DKU-20-045). The study was carried out in compliance with the ARRIVE guidelines.

### Histology analysis

The fixed skin tissues were moved to a tissue cassette and washed with PBS buffer to remove the rest of PFA. The samples were then dehydrated with a series of ethanol solutions (70–80–90–100%) and incubated with xylene. After embedding the samples in paraffin, the tissue blocks were sectioned into 6 μm thickness using a microtome (RM2255, Leica, Bensheim, Germany) and attached to tissue slides. The tissue slides were deparaffinized in xylene and then washed with a series of ethanol to perform hematoxylin–eosin staining. After the staining, the slides were dehydrated by ethanol and became transparent by xylene. Then, the cover glass was put on the slides using the mounting solution (Tissue-Tek^®^Glas™ Mounting Media, Sakura, Japan). The skin tissue slides were images and analyzed by a slide glass scanner, Easy Scan (Motic, Richmond, Canada).

### Statistical analysis

The mean and standard deviation of the mean were calculated from the data obtained with at least three replicates. An unpaired t-test and one-way analysis of variance (ANOVA) were utilized to determine the statistical significance in comparing ISF extraction differences between TMICS, SMICS, and SMN patch. In all data, a p-value < 0.05 was considered statistically significant. A p-value < 0.05, < 0.01, or < 0.001 was marked as one asterisk (*), two asterisks (**), or three asterisks (***) in the graphs.

## Supplementary Information

Supplementary Figures.
