# Supplementary material for: Enhanced extraction of skin interstitial fluid using a 3D printed device enabling tilted microneedle penetration
Source: Sci Rep. 2021 Jul 7;11:14018. doi: 10.1038/s41598-021-93235-3 (PMC8263571; doi:10.1038/s41598-021-93235-3)
Supplement: Supplementary file 1 — Supplementary Figures. [file 41598_2021_93235_MOESM1_ESM.pdf]

Supplementary Information

Enhanced extraction of skin interstitial fluid using a 3D printed device enabling tilted microneedle penetration

*Sanha Kim[1], Min Suk Lee[2], Hee Seok Yang[2,3], and Jae Hwan Jung[1]**

[1] Department of Pharmaceutical Engineering, Dankook University, 119 Dandae-ro, Dongnam-gu, Cheonan, 31116, Republic of Korea

[2] Department of Nanobiomedical Science & BK21 FOUR NBM Global Research Center for Regenerative Medicine, Dankook University, Cheonan 31116, Republic of Korea

[3] Center for Bio-Medical Engineering Core-Facility, Dankook University, Cheonan, 31116, Republic of Korea

*Corresponding author. E-mail: jjaehwan@dankook.ac.kr

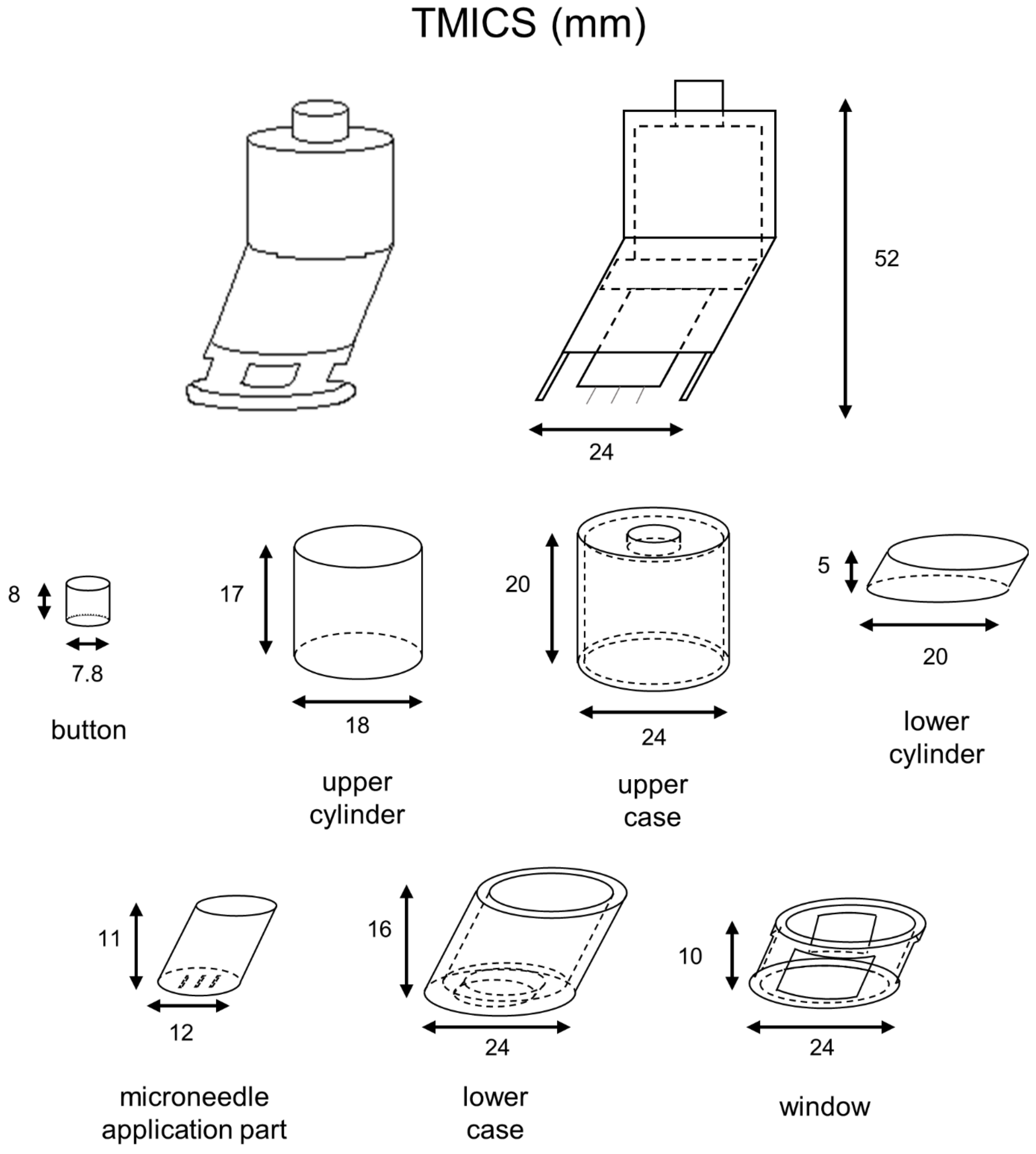

Fig. S1: CAD design of TMICS components. Design and specification outside and inside of the assembled TMICS and individual parts: a button, a cylinder in upper case, an upper case, a cylinder in lower case, an MN application part, a lower case, the windows. (All values are in mm.)

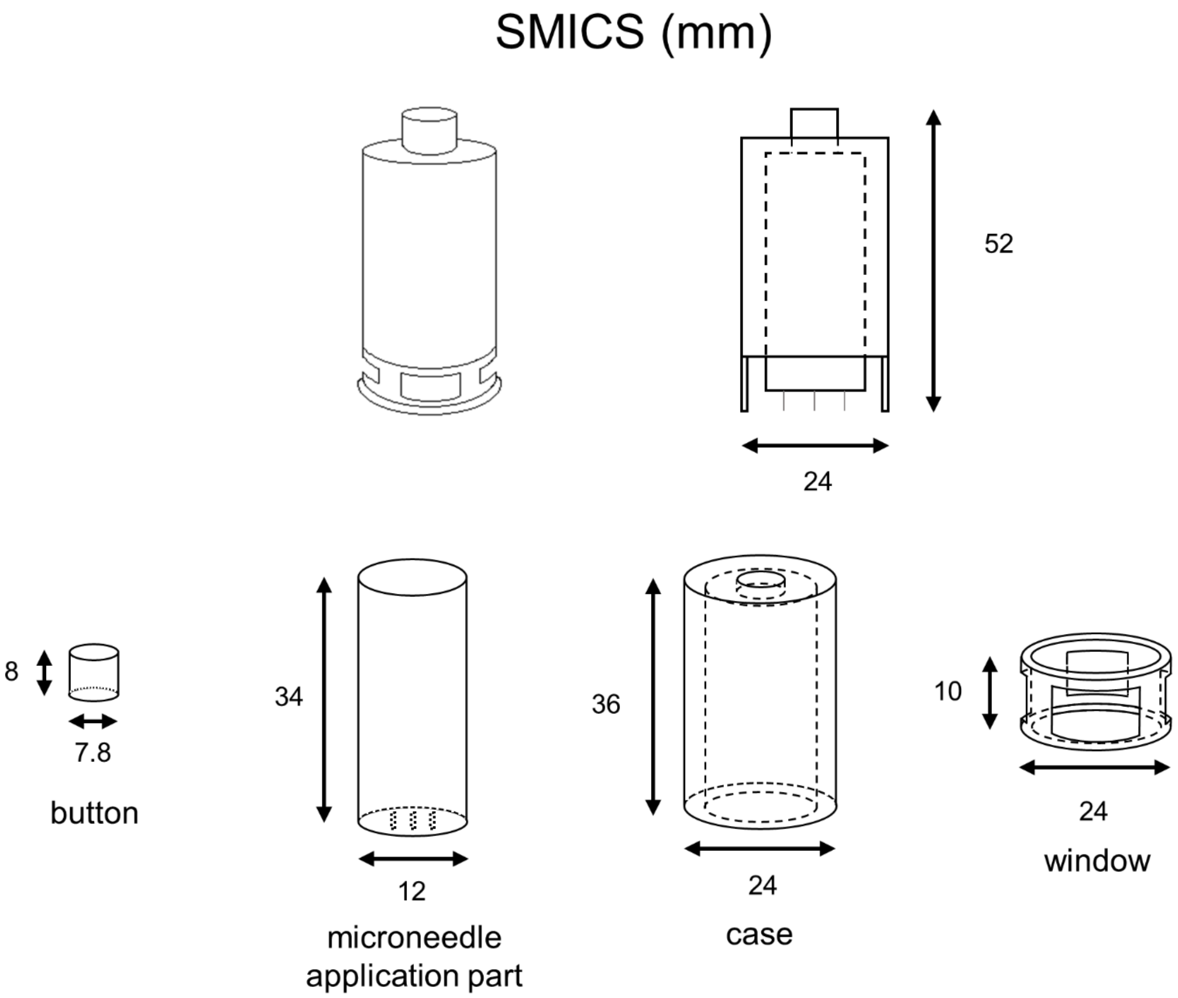

Fig. S2: CAD design of SMICS components. Design and specification outside and inside of the assembled TMICS and individual parts: a button, an MN application part in the case, SMICS case, the windows (All values are in mm.).

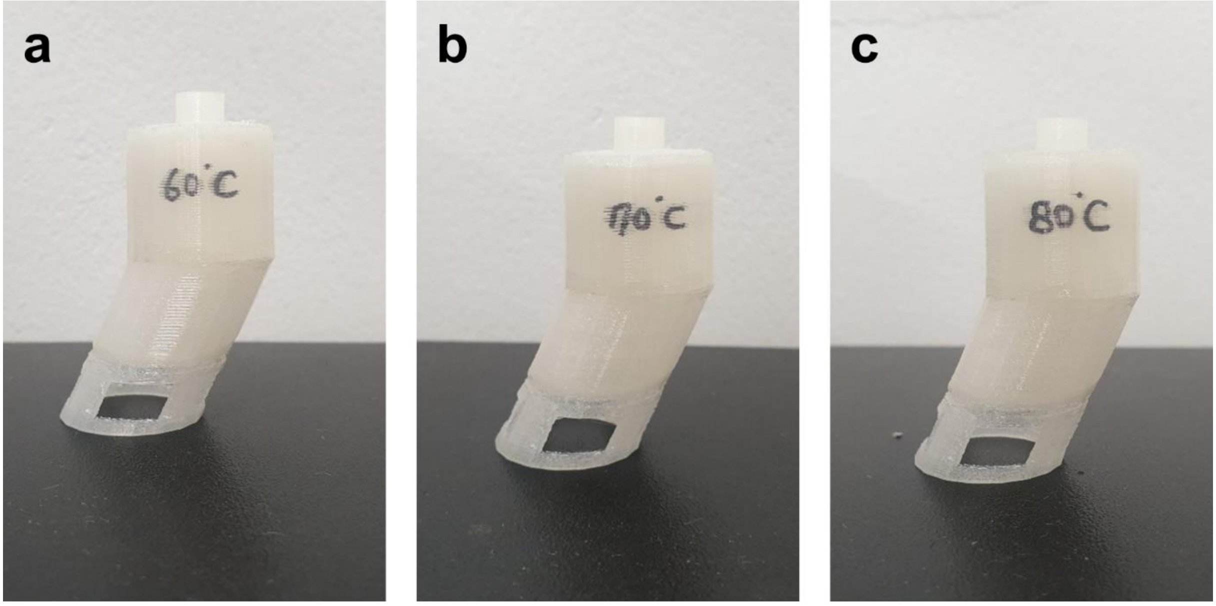

Fig. S3: Optimizing the temperature condition of the FDM 3D printing. The temperature of the heating bed was changed from 60 ℃ (a), 70 ℃ (b), and 80 ℃ (c) with maintaining the extruder temperature at 230 ℃.

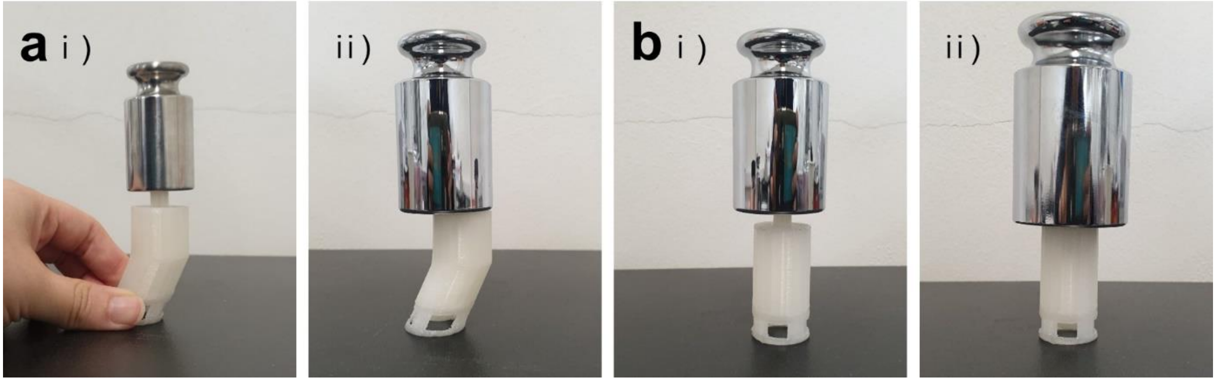

Fig. S4: Measurement of a minimal requiring force for the MN device operation. (a) TMICS operating test using a 200 g (i) and a 500 g (ii) weights. (b) SMICS operating test using a 500 g (i) and 1 kg (ii) weights. All the tests were repeated at least three times.

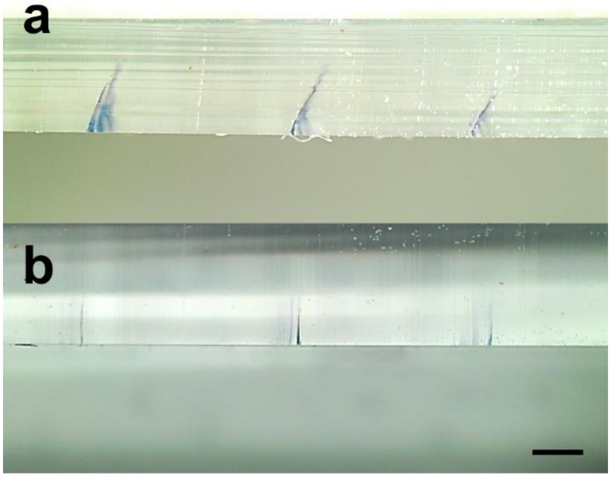

Fig. S5: Optical images of the MN trace that penetrate to PDMS artificial skin. (a) TMICS penetrates the MN at 66.3±0.8 degrees. (b) SMICS at 90.2±0.9 degrees. The scale bar is 500 μm.

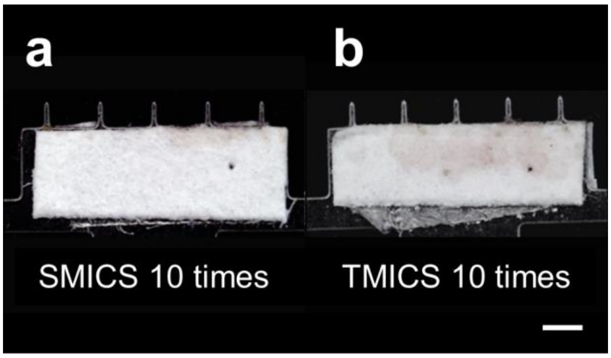

Fig. S6: Representative images of ISF collected in the paper reservoir from a rat in vivo skin. (a) ISF collected after applying SMICS 10 times (b) ISF collected after applying TMICS 10 times The scale bar is 1 mm.
